# Social cognitive mechanisms in healthcare worker resilience across time during the pandemic

**DOI:** 10.1007/s00127-022-02247-5

**Published:** 2022-02-26

**Authors:** Andrew J. Smith, Kotaro Shoji, Brandon J. Griffin, Lauren M. Sippel, Emily R. Dworkin, Hannah M. Wright, Ellen Morrow, Amy Locke, Tiffany M. Love, J. Irene Harris, Krzysztof Kaniasty, Scott A. Langenecker, Charles C. Benight

**Affiliations:** 1 Department of Psychiatry, Geisel School of Medicine at Dartmouth, Hanover, NH USA; 2 Department of Psychiatry, Huntsman Mental Health Institute, University of Utah School of Medicine, 501 Chipeta Way, Salt Lake City, UT 84018 USA; 3 Lyda Hill Institute for Human Resilience, Colorado Springs, USA; 4 University of Human Environments, Okazaki, Japan; 5 Mental Health Service, Central Arkansas VA Health Care System, Little Rock, USA; 6 Department of Psychiatry, University of Arkansas for Medical Sciences, Little Rock, USA; 7 VA Northeast Program Evaluation Center, West Haven, CT USA; 8 Department of Psychiatry, Geisel School of Medicine at Dartmouth, Hanover, NH USA; 9 National Center for PTSD, West Haven, CT USA; 10 University of Washington School of Medicine, Seattle, USA; 11 University of Utah, Resiliency Center, Salt Lake City, USA; 12 Bedford VA Healthcare System, University of Minnesota, Minneapolis, USA; 13 Department of Psychology, Indiana University of Pennsylvania, Indiana, USA; 14 Institute of Psychology, Polish Academy of Sciences (Poland), Warsaw, Poland; 15 Department of Psychology, University of Colorado-Colorado Springs, Colorado Springs, USA

**Keywords:** Resilience, Coping, Coping self-efficacy, Social support, PTSD, Traumatic stress, Pandemic, Disaster mental health, COVID-19, Healthcare workers, Frontline workers

## Abstract

**Purpose:**

Healthcare workers are at increased risk for mental health problems during disasters such as the COVID-19 pandemic. Identifying resilience mechanisms can inform development of interventions for this population. The current study examined pathways that may support healthcare worker resilience, specifically testing enabling (social support enabled self-efficacy) and cultivation (self-efficacy cultivating support) models.

**Methods:**

Healthcare workers (*N* = 828) in the Rocky Mountain West completed self-report measures at four time points (once per month from April to July of 2020). We estimated structural equation models to explore the potential mediating effects that received social support and coping self-efficacy had (at time 2 and time 3) between traumatic stress symptom severity (at time 1 and time 4). Models included covariates gender, age, minority status, and time lagged co-variations between the proposed mediators (social support and coping self-efficacy).

**Results:**

The full model fit the data well, CFI = .993, SRMR = .027, RMSEA = .036 [90% CIs (0.013, 0.057)]. Tests of sequential mediation supported enabling model dynamics. Specifically, the effects of time 1 traumatic stress severity were mediated through received social support at time 2 and time 3 coping self-efficacy, in sequential order to reduce time 4 traumatic stress severity.

**Conclusions:**

Findings show the importance of received social support and coping self-efficacy in mitigating psychopathology risk. Interventions can support mental health by focusing on social resource engagement that facilitates coping empowerment, which may decrease risk for mental health job-related problems among frontline healthcare workers exposed to highly stressful events.

**Supplementary Information:**

The online version contains supplementary material available at 10.1007/s00127-022-02247-5.

## Introduction

At the outset of the novel coronavirus (COVID-19) pandemic, more than 50% of frontline workers reported increased risk for stress-related syndromes such as posttraumatic stress disorder (PTSD), depression, anxiety, problematic alcohol use, and insomnia [22, 73, 83, 103]. These increased risks add to perennial mental health problems among healthcare workers that preceded the pandemic, exemplified by high rates of burnout, job turnover, psychiatric distress, and suicide risk [18, 40, 55, 71, 72]. A recent meta-analytic review revealed high risk for post-pandemic PTSD among health care workers (26.9%; [104]).

Research is needed to identify factors that facilitate resilience and mitigate development and persistence of psychopathology [38, 61]. Identifying mechanisms of resilience can inform preventative stress coping strategies, as well as targets in formal and informal interventions across clinical, organizational, and public health settings [16, 39, 65].

## Resilience via the interplay of interpersonal and intra-individual processes

Resilience is defined as the ability to adapt or “bounce back” in healthy ways from adversity, trauma, threat, and/or tragedy, and is a dynamic process determined by complex bio-psycho-social capacities [see 38, 87]. Notwithstanding complexities that will influence resilience in the COVID-19 pandemic context [38], engagement with social resources (in individual relationships, organizations, communities) and positive interpersonal interactions are critically important for resilience and human thriving [21, 56, 66, 78, 86]. They are especially important during and after traumatic stress exposures [24, 100], with evidence suggesting that they predict recovery in collective/community trauma settings (a traumatic event[s] such as a pandemic or natural disaster that affects an entire people group or society; [see 28, 80, 81]), and are integrally associated  with PTSD symptoms over time [101]. In clinical settings, social support influences patients’ ability to extract benefits from trauma-focused treatments [12, 45, 58, 76]. Cross-sectional research conducted during the COVID-19 pandemic identified social support as a predictor and buffer of mental health among healthcare workers [31] and community members [43], and likely to be involved in development, persistence, and/or resolution of post-pandemic PTSD [104].

While strong evidence indicates that social resources play a critical role in resilience, application of this knowledge to development and optimization of preventive interventions requires the study of “how” we extract social support benefits and prevent harms. “How” social environments influence resilience is a central focus of Social Cognitive Theory of Stress Adaptation, a framework built on two major tenets [6].

First, self-regulation is a function of the interaction among behavior, the person, and the environment where self-referent appraisals drive adaptation [2, 3, 6]. Self-regulation is largely driven through self-evaluative feedback forming coping self-efficacy (CSE) perceptions. CSE is defined as the belief about one’s ability to manage the demands of a stressful or traumatic environment (e.g., “I am capable of managing my emotions when I am reminded of the trauma”) [6]. Higher CSE exerts direct effects on regulating biological stress reactions [1, 93], thereby conserving internal resources that can be applied to higher order functioning and goals. Additionally, higher CSE (about one’s ability to respond adaptively to stress) increases the likelihood of initiating successful coping behaviors, which further reinforces one’s beliefs about CSE. Meta-analyses of studies conducted in diverse settings support CSE as predicting large effects on resilient adaptation and posttraumatic health [4, 47, 75].

A second major tenet of Social Cognitive Theory of Stress Adaptation is that social environments interact with self-regulation processes [2, 6, 69]. Social resources (e.g., received social support) provide stress regulation benefits, both through direct downregulation of sympathetic nervous system arousal [14, 15, 49, 51] and through transaction with one’s CSE appraisals [69, 74]. Recovering from exposure to trauma can exceed individual coping capabilities, requiring us to seek and utilize social resources to boost coping success [80, 100]. Experiences with supportive and/or aversive relationships are evaluated cognitively, through our perceptions of how capable we are to successfully respond to posttraumatic demands and initiate adaptive behavioral actions (“even though my distress is high today, I can go to work and do my nursing job because my spouse and/or co-worker will help me manage life and work demands”) (see [100]).

## Enabling and cultivation models of resilience

This resilience-promoting interplay between internal coping appraisals and external social resources is proposed to occur through two mechanisms: (1) social interactions that enable, empower, or improve an individual’s CSE perceptions (i.e., enabling hypothesis), and/or (2) higher CSE beliefs that enhance one’s ability to extract the health-promoting qualities available in social relationships (i.e., cultivation hypothesis, [69]). Evidence supports the enabling hypothesis in military veterans, community-level collective trauma settings, and among patients in a clinical setting undergoing trauma-focused therapy [76, 79, 80]. Interestingly and particularly relevant to the present study, evidence also supports the cultivation hypothesis among healthcare providers [74]. Both hypotheses have support among cancer patients [4, 30].

## The current study

Given that the enabling hypothesis has been supported in a collective trauma setting (mass community violence; [80]), and the cultivation hypothesis has been supported among healthcare providers in an individual trauma setting (i.e., secondary traumatization; [74]), it is unclear which hypothesis would be supported in a combination of these conditions (i.e., healthcare providers in a collective trauma setting such as the pandemic). No study to date has examined this question. As such, this study tested enabling and cultivation hypotheses of resilience among healthcare workers in the COVID-19 pandemic, whom we assessed across four time points (once per month from April through July of 2020).  We examined enabling and cultivation hypotheses in a manner that tests causal inferences while also modeling time-lagged effects for how the proposed mediators (social resources and CSE) affect one another  while controlling for their respective effects from previous time points. This type of modeling has only been used to test enabling and cultivation hypotheses in one previous study conducted in a context that is distinct (i.e., among cancer patients, [30]) from the current study setting.

The primary research question was: do enabling and/or cultivation dynamics at times 2 and 3 (May 2020, June 2020) mediate the effects of time 1 traumatic stress symptom severity (April 2020) on time 4 traumatic stress symptom severity (July 2020)? We hypothesized that enabling and cultivation pathways would work as mechanisms of resilience by mediating the effects of time 1 traumatic stress symptoms on time 4 traumatic stress symptom severity. Our a-priori hypothesis about which pathway would emerge as significant was exploratory, because the literature supports the enabling hypothesis in a collective trauma setting [80] and the cultivation hypothesis with healthcare workers in an individual trauma context [74].

## Methods

### Participants

**Fig. 1:**
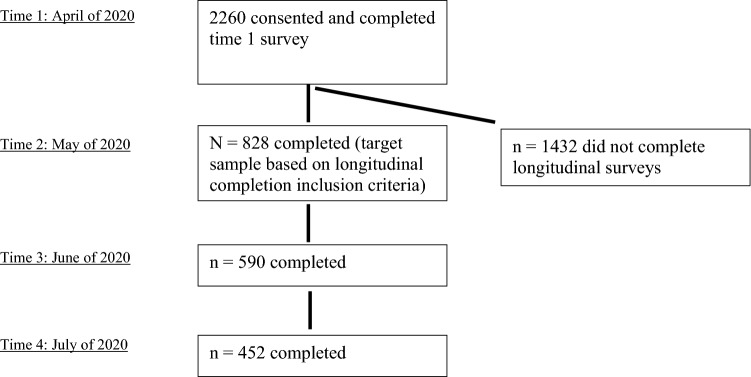
Participation across four time points of survey data collection

Participants for this study included 828 healthcare professionals working in a large healthcare system in the Rocky Mountain Western United States (mean age = 39.54 years old [SD = 12.55], 81.76% female) who met study inclusion criteria by completing measures at time 1 (T1) and time 2 (T2). These 828 participants are from an initial cross-sectional survey completed by 2,260 (see [83]). Participants were sent the initial survey link by their administrative leadership through a listserv. A total of 22,540 healthcare staff received the email containing the survey link, 13,817 opened the email, and 2,988 opened the embedded survey link and consented to participate. Data cleaning led to removal of 728 cases, leaving 2,260 participants at T1 (see [84]). At the end of the initial T1 survey, participants were asked if they would agree to be assessed at additional time points, which required a “yes” consent and entry of an email address, to which survey links were sent 30 days after completion of the previous survey across four months. See Fig. 1 for reporting of timing of survey administrations and participation across four study time points.

At T1 (*N* = 828) participants included 92.51% Caucasian, 3.02% Asian, 0.72% Native American/Alaskan Native, 0.60% African American, 0.12% Native Hawaiian/Pacific Islander, and 4.23% other ethnicities. Their roles included nurse (39.27%), clinical staff (technician, therapist; 20.97%), attending physician (7.65%), resident/fellow (6.66%), advanced practice clinician (3.16%), pharmacy professional (4.16%), mental health professional (5.32%), and other (12.81%). The majority of healthcare professionals endorsed that they function in a ‘direct patient care’ role (72.85%). Average length of career was 10.43 years (SD = 9.44). Demographics of this sample proved similar to those observed in the U.S. Census [98] among health care workers.

### Measures

#### Traumatic stress symptoms

We assessed traumatic stress with an adapted Primary Care PTSD Screen for DSM-5 (PC-PTSD-5), including five items measuring severity of symptoms during the past month [60]. Sample items include “had nightmares about the event(s) or thought about the event(s) when you did not want to?” and “tried hard not to think about the event(s) or went out of your way to avoid situations that reminded you of the event(s)?” The original measure uses a yes–no response format, which we adapted to a 5-point scale (0 = *not at all*, 4 = *extremely*; borrowed from the Posttraumatic Stress Disorder Checklist for DSM-5, [9]). Moreover, participants were asked to complete the measure whether or not they endorsed a significant history of criterion A traumatic event exposure. Internal consistency was high for the Likert-scale adapted version, Cronbach’s *α*’s of 0.83 (T1) and 0.87 (T4). In addition to calculating mean scores, we computed dichotomous scores. We considered a score of 2 or higher (moderate to extreme severity for that item) to be a “yes” response (indicating a clinically significant symptom endorsement), and collapsed each item response into a binary score (0 = no or minimal symptom severity; 1 = clinically significant at “moderately” or higher). We calculated sum scores by aggregating the dichotomized responses, and scores ranged from 0 to 5 (with the clinical significance cutoff point of 3 or higher, see [60]).

#### Coping self-efficacy

We assessed perceived ability to cope with challenges and uncertainties during the pandemic using the Pandemic CSE Scale at T2 and T3. We created this measure by adapting items from other CSE measures [8]. Five items comprised the scale, to which participants responded using a 7-point response format (1 = *not capable* to 7 = *very capable*). These items begin with a stem, “In the Coronavirus pandemic, how capable are you to…”. Sample items included “deal with my emotions (anger, sadness, depression, anxiety) since the pandemic,” “keep my life feeling normal,” and “manage distressing images or dreams about the pandemic.” We aggregated the items into a sum total score. This scale had high internal consistency, Cronbach’s alpha = 0.88 (T2) and 0.89 (T3).

#### Received social support

We used four items to assess social support received from four sources (family, friends, co-workers, and community members) at T2 and T3, which we adapted from previous clinical research [17]. The following stem was used: “On a scale 0 to 10, with 10 being total support and 0 being no support, how much do you feel you get from the following sources since the start of the COVID-19 pandemic?” We aggregated all four items into a sum score, supported by adequate internal consistency, Cronbach’s alphas of 0.77 (T2) and 0.80 (T3).

### Procedures

#### Study design

The outcome variable for this study was T4 traumatic stress symptom severity, and independent variables were T1 traumatic stress symptoms, T2 received social support and CSE, and T3 received social support and CSE.

#### Data handling

Among the sample at T1 and T2 (*N* = 828), there were 0.37% of missing data. There were 0.45% of missing data among 590 participants who completed the T3 questionnaires, and there were 0.32% of missing data among 452 participants who completed the T4 questionnaires. To evaluate patterns of missing data, we conducted tests of missing completely at random (MCAR) using the methodology proposed by Jamshidian and Jalal [33]. MCAR refers to the probability of missingness not depending on either observed variables nor unobserved variables [67]. Results of the MCAR test showed a significant Hawkins test (*p* < 0.001), but the non-parametric test was not significant (*p* = 0.230), indicating that the missing data for T2 were MCAR. For T3, the Hawkins test was significant (*p* < 0.001), but the non-parametric test was not significant (*p* = 0.285), indicating MCAR. For T4, the Hawkins test was significant (*p* < 0.001), but the non-parametric test was not significant (*p* = 0.079), indicating MCAR. The missing data were imputed for each time point with the SVDimpute algorithm using an R package pcaMethods [88, 95].

#### Analyses

Preliminary analyses included a series of MANOVAs to test for potential differences based on attrition. Primary analyses involved a series of structural equation models (SEM) to evaluate the enabling and cultivation hypotheses using an R package lavaan [63]. First, we tested a model in which T1 traumatic stress symptom severity predicted T4 traumatic stress symptom severity directly and indirectly via CSE and received social support at T2 and T3 (Fig. 1). Covariates for T4 traumatic stress symptom severity included gender, minority status, and age. We also covaried for time lagged relationships within (i.e., CSE and received social support at T2, CSE and received social support at T3) and between (CSE at T2 and T3, received social support at T2 and T3) assessment occasions. The *a-priori* determined goal was to examine model data-fit, and to modify by removing pathways with *p* values greater than 0.1 if model fit was not adequate. We used a standard bootstrapping method to calculate standard errors with 5000 bootstrap samples. By using the standard bootstrapping method, we calculated coefficients and 95% bootstrap confidence intervals (CI) for indirect pathways. To evaluate the goodness-of-fit, we used comparative fit index (CFI) greater or equal to 0.95, standardized root mean square residual (SRMR) smaller than 0.08, and root mean square error of approximation (RMSEA) smaller than 0.07 as indicators for the good model fit [32, 89].

## Results

### Attrition analysis

We performed a series of tests to determine if dropouts and completers were different in the variables of interest at each time point (i.e., T1 traumatic stress and age; T2 traumatic stress, received social support, and CSE, and T3 traumatic stress, received social support, and CSE; T4 traumatic stress). We tested the differences between those who completed the T2 assessment (*n* = 828) and those who did not complete it (*n* = 1432), with a MANOVA, and found that the omnibus test was not significant, approximated *F*(2, 2147) = 2.96, *p* = 0.052. Next, we tested whether there was a difference between those who completed T3 assessment (*n* = 590) and those who did not (*n* = 238) with a MANOVA was performed on T2 CSE, T2 received social support, and T2 traumatic stress. Results showed that there were no significant differences between T3 completers and dropouts, approximated *F*(3, 824) = 0.22, *p* = 0.879. A MANOVA was conducted to test differences between those who completed the T4 assessment (*n* = 452) and those who did not (*n* = 138) on T3 CSE, T3 received social support, and T3 traumatic stress. No significant differences were shown between T4 completers and dropouts, *F*(3, 586) = 0.26, *p* = 0.851. Finally, we tested whether high traumatic stress severity was related to attrition. Specifically, a one-way ANOVA was employed to examine whether there was a significant difference in T1 traumatic stress severity between those who dropped out between T1 and T4, and those who participated across time. Results showed no significant difference in T1 traumatic stress severity between those who dropped out (*M* = 1.33, *n* = 376) those who participated (*M* = 1.27, *n* = 452), *F*(1, 826) = 1.11, *p* = 0.293, Cohen’s f = 0.037.

### Correlations among primary variables

Table 1 displays a correlation matrix for the relationships among these variables. Importantly, traumatic stress symptom severity at T1 and T4 were negatively related to CSE and received social support at T2 and T3 with small to large effect sizes (*r* range = − 0.60 to − 0.23). CSE at T2 and T3 were positively related to received social support at T2 and T3 with medium effect sizes (*r* range = 0.36 to 0.45). Risk threshold for possible PTSD was met for approximately 37.08% of participants at T1 and 29.20% at T4 (i.e., PC-PTSD score of 3 or higher, see [60]). Note that our PTSD risk rates were based on an adapted PC-PTSD measure, and that the PC-PTSD measure is a screening tool; the gold standard for PTSD diagnostic determination was not utilized in the current study (i.e., the Clinician Administered PTSD Scale; [102]). The possible PTSD diagnostic risk rates that we evaluated should be interpreted with caution.

**Table 1:** Means, standard deviations, and correlations with confidence intervals

Variable	M	SD	1	2	3	4	5	6
1. T1 TSS	1.30	0.88						
2. T4 TSS	1.13	0.90	0.67**					
3. T2 CSE	5.05	1.20	− 0.56**	− 0.55**				
4. T3 CSE	5.00	1.23	− 0.52**	− 0.60**	0.74**			
5. T2 RSS	6.44	2.03	− 0.23**	− 0.26**	0.42**	0.36**		
6. T3 RSS	6.14	2.13	− 0.23**	− 0.31**	0.41**	0.45**	0.71**	
7. Age	39.98	11.89	− 0.14**	− 0.20**	0.18**	0.18**	0.10**	0.10*

*M* and *SD* are used to represent mean and standard deviation, respectively

*M* mean, *SD* standard deviation, *TSS* traumatic stress symptoms, *CSE* coping self-efficacy, *RSS* received social support

*Indicates *p* < 0.05. **Indicates *p* < 0.01

### Testing the enabling and cultivation hypotheses

**Fig. 2:**
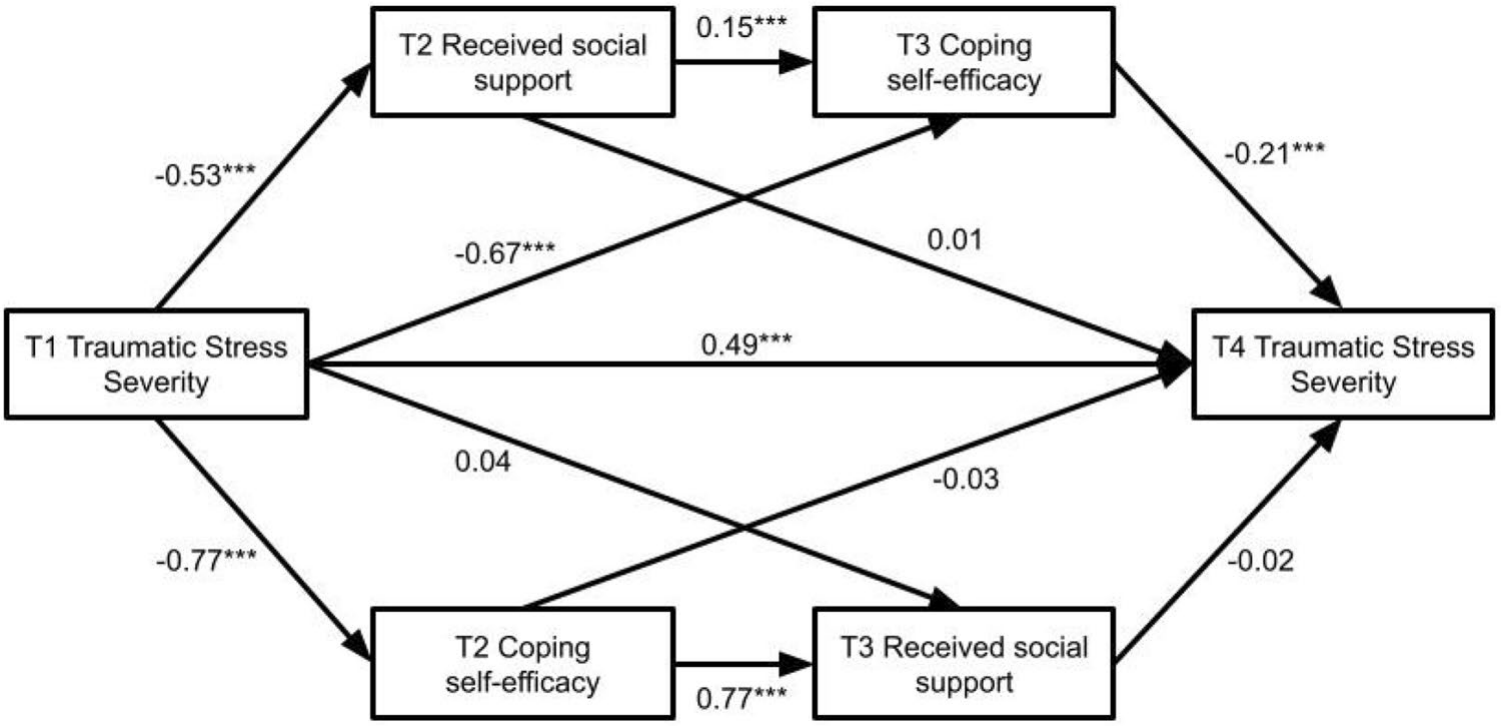
Unstandardized coefficients for the cultivation and enabling models with FIML. Covariates, covariances, and variances are omitted from the figure for the clarity. Unstandardized coefficients are 0.00 (*p* = 0.964) for the relationship between gender and T4 traumatic stress severity, 0.11 (*p* = 0.416) for the relationship between minority status and T4 traumatic stress severity, and − 0.01 (*p* = 0.045) for the relationship between age and T4 traumatic stress severity. Variances are 3.93 for T2 received social support, 0.99 for T2 coping self-efficacy, 3.66 for T3 received social support, 1.02 for T3 coping self-efficacy, and 0.39 for T4 traumatic stress severity. Covariances are 0.57 for the relationship between T2 and T3 coping self-efficacy (*p* < 0.001), 2.28 for the relationship between T2 and T3 received social support (*p* < 0.001), 0.71 for the relationship between T2 received social support and T2 coping self-efficacy (*p* < 0.001), and 0.01 for the relationship between T3 received social support and T3 coping self-efficacy (*p* = 0.942). *** indicates *p* < 0.001

See Fig. 2 for a summary of the structural equation model that was tested. Structural equation modeling included the variables and pathways for both the enabling hypothesis and cultivation hypothesis using FIML to manage missing data (*N* = 828). FIML cannot be applied to exogenous variables, and as such, we excluded participants who did not have data for the covariates. After excluding 61 participants who did not respond to the questions for the covariates (age, minority status, or gender), the final model included 767 participants. Simulation studies on missing data imputation methods showed that FIML is more suitable to manage data with as much as 25% of missing data compared to other missing data management methods such as listwise and pairwise deletion [11, 68] and produces tolerable bias in results with up to 50% missingness in some cases even though standard errors tend to inflate as the proportion of missing data increases [42, 44]. The present study contained up to 41% missing data, the patterns of missing data indicated MCAR, suggesting that FIML can provide less biased results compared to results based on listwise deletion. Results indicated that the model-data fit was excellent, CFI = 0.993, SRMR = 0.027, RMSEA = 0.036 (90% CIs [0.013, 0.057]). Figure 2 shows the coefficients for the model.

#### Mediation/indirect effects

We tested the enabling hypothesis with a sequential indirect effect of T2 received social support and T3 CSE in mediation path between T1 traumatic stress symptom severity and T4 traumatic stress symptom severity. This mediation pathway was significant [*B* = 0.016, SE = 0.01, 95% bootstrap CIs (0.008, 0.028)], supporting the enabling hypothesis. We also tested the alternative cultivation hypothesis with a sequential effect of T2 CSE and T3 received social support in the pathway mediating between T1 traumatic stress symptom severity and T4 traumatic stress symptom severity. This sequential indirect effect was not significant [*B* = 0.014, SE = 0.01, 95% bootstrap CIs [− 0.012, 0.040)], failing to support the cultivation hypothesis.

Finally, to test the stability of the model, we conducted a parallel test of the full model with participants who completed the T4 questionnaire without using FIML (*N* = 452), which revealed that the FIML model findings were stable and consistent.^1^

## Discussion

The current study tested social cognitive models of resilience among healthcare providers during the first wave of the SARS-CoV-2 pandemic. We focused on resilience mechanisms that may be amenable to change via informal and formal interventions to disrupt the development, persistence, and/or exacerbation of traumatic stress symptoms by testing mediational effects of interpersonal social transactions (received social support) and intra-individual self-regulatory coping appraisals (CSE). These pathways were formulated using enabling and cultivation hypotheses [69], and mediation was modeled between T1 traumatic stress severity (April of 2020) and T4 traumatic stress symptom severity (July of 2020).

Results indicated that the full model that included the predictors of interest (T1 traumatic stress symptoms, T2 received social support and CSE, T3 received social support and CSE) and covariates (age, gender, race; time lagged co-variations between the proposed mediators) fit the data well. This overarching finding for adequate model fit supports social cognitive theory of posttraumatic adaptation [6] suggesting that the modeling of dynamics between interpersonal/social transactions and intra-individual self-regulatory coping is helpful in conceptualizing stress and coping processes among healthcare workers during the pandemic.

Results supported the enabling model of resilience, shown by the following dynamics: lower T1 traumatic stress symptoms were associated with higher T2 received social support; higher T2 social support was associated with higher T3 CSE; higher T3 CSE was associated with lower T4 traumatic stress symptom severity. Support for the enabling model is consistent with previous studies in non-clinical samples of military personnel and collective-trauma survivors [79, 80]. Although causality cannot be concluded given the observational design, these findings illuminate one potential way in which engagement with social relationships (family, friends, co-workers, community members) may promote resilience among non-clinical samples—by bolstering the individual’s perceived ability to cope (cognitive self-regulation).

Our study did not support the cultivation model. Although T2 CSE was related to receiving more social support, T3 received social support was not associated with lower T4 traumatic stress symptoms. Studies of actual receipt of help after collective upheavals often produce inconsistent findings concerning the benefits of received social support on survivors’ well-being [37]. For example, Platt et al. [57] showed specifically that emotional received support was associated with lower levels of posttraumatic stress, whereas tangible and informational received support sub-factors yielded null effects. On the other hand, previous research shows support for the cultivation model (vs. enabling model) under conditions of individually experienced events/situations (e.g., medical trauma, [30]; secondary traumatic stress, [74]). Individually experienced events can yield particularly complex social support seeking and disclosure challenges which involve the trauma exposed individual attempting to extract support from social network members who do not share in the stress/trauma experience (see [19]). The individual in these types of recovery environments is tasked to self-regulate at a higher emotional load level (using CSE appraisals) in order to extract social benefits that are critical for recovery amidst potentially confusing and invalidating responses from network members who may simply may not “get it,” and who may even communicate defensive and ambivalent messages.

Notably, in our study, we measured perception of social support received (rather than perceived availability of support), a measurement distinction that may have played a role in our different findings than previous research among healthcare workers [74]. Previous research shows that ‘receiving’ support may impact distress through CSE appraisals [77] and may have less direct impact on mental health outcomes than perceived social support does [53].

### Limitations

This is a study of a non-clinical population, and may not generalize to clinical settings. Additionally, our sample was predominantly female at ~ 82%. Although gender did not have a statistically significant relationship with the primary outcome of interest (time 4 traumatic stress), generalization of model findings to male healthcare providers is cautioned. Further, our social support measure is limited in depth and specificity based on our priorities to minimize survey length and participant burden in the midst of an ongoing disaster. Better measures of received social support should be incorporated in future research to more rigorously test the dynamic interplay of social support and coping during and after stressful events [20, 34, 63].

Our use of the adapted PC-PTSD-5 measure for assessing traumatic stress symptom severity is not tested psychometrically in the literature. We made adaptations intentionally to increase the utility of this measure in a nonclinical general population, for goals to (a) reduce risk for inflating the diagnostic rates with false positives during the acute phase and (b) increase variability for use as a continuous measure of severity. Further, we adapted participation logic compared to use in clinical settings by having participants complete the PC-PTSD-5 whether or not they endorsed a significant history of trauma. As such, we did not measure “PTSD symptoms,” but rather “traumatic stress symptoms” in the midst of an ongoing collective trauma. Moreover, we considered 3 or higher on the PC-PTSD-5 scale to be indicative of clinically significant symptoms, consistent with the currently accepted convention [60]; recent evidence suggests that it may be more accurate and useful to consider a higher cutoff score  [10]. Gold standard assessment instruments (e.g., Clinician Administered PTSD Scale; [102]) were not used due to the observational nature of this study. Altogether, interpretation of our diagnostic rates should be considered with caution based on these adaptations and caveats, and future psychometric testing of an adapted version such as the one we employed is needed.

A poignant limitation is associated with the racial homogeneity of our sample (~ 90% White), especially considering the disproportionate ways that COVID-19 affected persons of color [59, 64]). Research that does not represent persons of color risks continuing to exacerbate ongoing inequities in the medical system (see [23]). We incorporated a control variable (dummy coded white vs. non-white), with no indication that race played a role in our models. Nonetheless, research with persons of color should be prioritized.

## Conclusions

Our findings specifically highlight the importance of received social support in the enablement of coping and resilience during the COVID-19 pandemic. Having high distress and/or high intensity/severity of trauma experiences could have erosive effects on social support and interpersonal networks [35, 41, 46, 70]. Research on potentially traumatic events shows that social support can be difficult to extract, and social harms can be difficult to avoid in part due to misguided, ineffectual, or even negative or invalidating reactions from members of their social networks. These negative reactions may disrupt help seeking, disclosure willingness, emotion processing, received support that is extracted, and the degree to which support is perceived positively, which consequently interfere with longer-term recovery [36]. It is important to study such dynamics across longer periods of time [35, 46] and to identify possible complications for stress adaptation among healthcare workers who experience social rejection and neglect from fellow healthcare workers, organizations in which they are working, and within their larger social networks outside of work.

On the other hand, previous research in a community/collective trauma setting indicated that higher acute traumatic distress may serve as a “signal” of the need to engage in social support [90], which in-turn enhances CSE and reduces distress across time [80, 91]. It may be useful to consider ways to engineer social environments in individual, occupational, and community networks in ways that make social interactions more accessible (in-person, virtual, via telehealth technologies). Moreover, it may be useful to prompt individuals who are immersed in collectively experienced traumatic events (e.g., the pandemic, mass violence event, natural disaster) to intentionally engage in diverse types of social interactions.

The scope of the pandemic has laid bare the tremendous need for increased access to effective mental health support across a broader spectrum of populations (from generally healthy populations to those with severe mental health concerns). Community interventions that are easily accessible and bolster naturalistic coping resources (e.g., social support and CSE) are critical to increase access to useful preventative interventions. More people across a more diverse spectrum of functioning are in need of mental health support at the same time than we have seen previously, and we do not have the formal resources to meet this demand. Avenues for access to culturally palatable interventions that can be administered at scale are important to meet the needs of healthcare workers. This requires innovation beyond interventions that are administered one-on-one between clinician and client, beyond pathology-based approaches, towards preventative and well-being-oriented approaches [7].

For example, RE-WIRE (Re-engaging in Worthy Interpersonal Relationships) focuses on proactive engagement in supportive social relationships whether or not a client or group member has a mental health diagnosis, allowing for use in wellness/prevention settings and pathology treatment settings alike [84]. RE-WIRE targets social relationship improvement (not pathology) as a primary goal, doing so by making use of intervention tools and philosophies that are publicly available and interdisciplinary in nature [e.g., motivational interviewing; 50]. Another example is “Health and Strength,” a group-level intervention designed to increase access and interpersonal support for healthcare workers. Health and Strength was adapted from a pre-existing intervention [“Building Spiritual Strength,” 25, 26, 27, 96, 97], and uses a psychospiritual developmental model to help healthcare workers resolve distressing emotions associated with the ethical dilemmas that they have experienced throughout the COVID-19 pandemic. Community level intervention programs such as GRIT (Greater Resilience Information Toolkit) represent key innovation as well. GRIT was designed to empower the social system of the community or healthcare unit to respond to threatening and changing traumatic environmental demands, to increase the care that communities and social networks can naturally provide to raise the baseline level of resilience [6, 29, 47, 48]. Research on the efficacy of these novel interventions (and others like them) is important, with a particular focus on how these interventions target and improve mechanisms of interpersonal connectivity and self-efficacy.

There are diverse social resources upon which healthcare workers can capitalize in the long-term adaptation process. We must be attuned to risks for negative social feedback loops which individuals in high distress or trauma exposed states are particularly prone to experiencing; such negative feedback loops can diminish access and efficacy of natural coping resources. Being in supportive relationships with other healthcare workers who have shared experiences is likely to be important in the recovery process [91, 92]. Beyond engaging with fellow healthcare workers, engagement in support with non-healthcare workers may also be critical, and can be pursued based on shared meaning and stressful pandemic experiences that transcend work environments (e.g., parenting stressors; school stressors; scarcity of resources; loss of routine; increase in stress/tension at home; grief and loss). Such diversity of support options is likely to be critical across time [81], as professional identity is not the only valid or healthy source of identity, meaning, or connection. Our multitude of roles (mother, father, sister, brother, parent, child, hobbies from which we derive identity capital) each offer inroads to sharing experiences and extracting support in meaningful ways that can promote resilience and well-being.

## Supplementary Information

Below is the link to the electronic supplementary material.

Supplementary file1 (DOCX 103 KB)

## Footnotes

1 See supplemental Fig. 1 for the model tested using listwise deletion.
