# Correction to: Social cognitive mechanisms in healthcare worker resilience across time during the pandemic

**DOI:** 10.1007/s00127-022-02275-1

**Published:** 2022-03-21

**Authors:** Andrew J. Smith, Kotaro Shoji, Brandon J. Griffin, Lauren M. Sippel, Emily R. Dworkin, Hannah M. Wright, Ellen Morrow, Amy Locke, Tiffany M. Love, J. Irene Harris, Krzysztof Kaniasty, Scott A. Langenecker, Charles C. Benight

**Affiliations:** 1 Department of Psychiatry, Geisel School of Medicine at Dartmouth, Hanover, NH USA; 2 Department of Psychiatry, Huntsman Mental Health Institute, University of Utah School of Medicine, 501 Chipeta Way, Salt Lake City, UT 84018 USA; 3 Lyda Hill Institute for Human Resilience, Colorado Springs, USA; 4 University of Human Environments, Okazaki, Japan; 5 Mental Health Service, Central Arkansas VA Health Care System, Little Rock, USA; 6 Department of Psychiatry, University of Arkansas for Medical Sciences, Little Rock, USA; 7 VA Northeast Program Evaluation Center, West Haven, CT USA; 8 Department of Psychiatry, Geisel School of Medicine at Dartmouth, Hanover, NH USA; 9 National Center for PTSD, West Haven, CT USA; 10 University of Washington School of Medicine, Seattle, USA; 11 University of Utah, Resiliency Center, Salt Lake City, USA; 12 Bedford VA Healthcare System, University of Minnesota, Minneapolis, USA; 13 Department of Psychology, Indiana University of Pennsylvania, Indiana, USA; 14 Institute of Psychology, Polish Academy of Sciences (Poland), Warsaw, Poland; 15 Department of Psychology, University of Colorado-Colorado Springs, Colorado Springs, USA

## Correction to: Social Psychiatry and Psychiatric Epidemiology 10.1007/s00127-022-02247-5

The corresponding author Andrew J. Smith, PhD affiliations are as follows

^1^Department of Psychiatry, Geisel School of Medicine at Dartmouth, Hanover, NH, USA

^2^Department of Psychiatry, Huntsman Mental Health Institute, University of Utah School of Medicine, 501 Chipeta Way, Salt Lake City, UT 84018, USA

^3^Lyda Hill Institute for Human Resilience, Colorado Springs, USA

In the original version of the article, Andrew J. Smith affiliation was incorrectly printed.

The original article has been corrected.

