# Supplementary material for: Social cognitive mechanisms in healthcare worker resilience across time during the pandemic
Source: Soc Psychiatry Psychiatr Epidemiol. 2022 Feb 26;57(7):1457–68. doi: 10.1007/s00127-022-02247-5 (PMC8881189; doi:10.1007/s00127-022-02247-5)
Supplement: Supplementary file 1 — Supplementary file1 (DOCX 103 KB) [file 127_2022_2247_MOESM1_ESM.docx]

Appendix 1


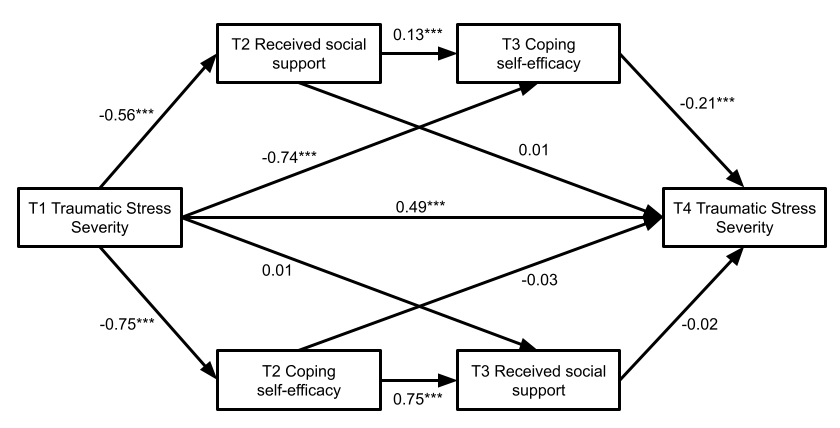


Unstandardized coefficients for the cultivation and enabling models with listwise deletion (N = 413).
*Note*. Covariates, covariances, and variances are omitted from the figure for the clarity. Unstandardized coefficients are 0.00 (p = .962) for the relationship between gender and T4 traumatic stress severity, 0.11 (p = .411) for the relationship between minority status and T4 traumatic stress severity, and -.01 (p = .052) for the relationship between age and T4 traumatic stress severity. Variances are 3.95 for T2 received social support, 1.02 for T2 coping self-efficacy, 3.81 for T3 received social support, 1.00 for T3 coping self-efficacy, and 0.39 for T4 traumatic stress severity. Covariances are 0.59 for the relationship between T2 and T3 coping self-efficacy (p < .001), 2.43 for the relationship between T2 and T3 received social support (p < .001), 0.65 for the relationship between T2 received social support and T2 coping self-efficacy (p < .001), and 0.07 for the relationship between T3 received social support and T3 coping self-efficacy (p = .474).

*** indicates p < .001.
